# Call for a Convention of All American Colleges

**Published:** 1879-03

**Authors:** 


### ARTICLE XV.

CALL FOR A CONVENTION OF ALL AMERICAN MEDICAL COLLEGES, to be held in the City of Atlanta, Ga., beginning at 10 A. M., Friday, May 2d, 1879.

*Editor Chicago Medical Journal Examiner.*

DEAR SIR: At the meeting of the "American Medical College Association," held in Buffalo, N. Y., June, 1878, Prof. S. D. Gross, a delegate from Jefferson Medical College, of Philadelphia, offered the following preamble and resolutions:

"WHEREAS, It is eminently desirable that the medical schools of this country should adopt a uniform system of instruction of a grade fully in accord with the requirements of the age in other branches of study, and with the practice of the medical institutions of Europe; and,

"WHEREAS, All the efforts to bring about such a change on the part of the American Medical Association, of the Association of Medical Teachers assembled at Cincinnati in 1867, and at Washington in 1869, and of different State medical societies, have signally failed, and,

"WHEREAS, The present time seems to be peculiarly favorable for taking strong ground upon the subject, inasmuch as it is now attracting general attention throughout the United States; therefore,

"*Resolved*, That this Association respectfully and earnestly request that the regularly organized and accredited medical schools of the United States hold, at their earliest convenience, a meeting for the purpose of adopting some definite and final action upon a subject of such vital importance to the dignity, character and usefulness of the profession, and the welfare of the American people.

"*Resolved*, That in order to impart proper efficiency to this plan, each and every college be requested to send two delegates, consisting of one member of each Board of Trustees, and one member of each Faculty, with full power to act for their respective institutions.

"*Resolved*, That the medical and secular press throughout the United States be respectfully requested to lend their aid in the dissemination and discussion of these preambles and resolutions, in order to place the whole matter of medical education prominently before the profession and the people.

"*Resolved*, That a copy of these preambles and resolutions, signed by the President and Secretary of this Association, be transmitted to the officers of every regularly constituted medical college in the United States, with a request to hold the contemplated meeting at Washington City, or at some other central point, on the first Wednesday in September next, or as soon thereafter as possible."

Prof. N. S. Davis seconded these preambles and resolutions, and heartily endorsed them.

Prof. T. G. Richardson moved that the time of the proposed meeting be fixed at the Friday preceding the meeting of the American Medical Association, and that the place be designated by the President of this Association. This amendment was adopted, and the preamble and resolutions as amended, were adopted.

In compliance with this action of the American Medical College Association, its acting President, Prof. N. S. Davis, of Chicago, has designated *Atlanta*, *Ga.*, as the *place* of the proposed convention, while the aforementioned action of the Association has fixed the *time* at 10 A. M. Friday, May 2d, 1879. It is earnestly hoped that delegates from all "regularly organized and accredited medical schools" in the United States, will promptly meet at the above designated time and place. That the action of the convention may be definite, it is desired that each college send two delegates, with full power to act for their respective institutions—one of these delegates to be selected from the Board of Trustees, and one from the Faculty.

In general terms, the *object* of the convention is to adopt some "uniform system of instruction more in harmony with the requirements of the age." Among the questions appropriate for discussion and decision may be memtioned, "Shall all the colleges require attendance upon three regular courses of lectures during three separate years, ere admitting students to become candidates for the degree of M.D.?

Is any uniform system possible in this or other things? If so, to what extent is it possible or even desirable at the present time? Each doctor in the land doubtless has in mind an ideal medical college system. But this convention cannot act upon idealities; it can only act upon that which is practicable to all honest efficient medical schools.

It is hoped that the medical press and teaching fraternity will freely and exhaustively discuss the subject matter of this convention. The doing of this at once will enable it to enter upon its labors with a very complete knowledge of the facts in the case.

To avoid misconception, let it be distinctly noticed that, although this convention is called by the "American Medical College Association," it is entirely distinct from that body. When assembled, the convention will elect its own officers and adopt its own methods for transacting its business in pursuance with the object of the call.

Signed, NATHAN S. DAVIS, M. D.,
*Acting President of the American Medical College Association.*

LEARTUS CONNOR, M. D.,
*Secretary, Detroit, Mich.*

FEBRUARY 6TH, 1879.

P. S.—It is desired that those colleges which decide to attend the aforesaid convention by delegates, will send notice to that effect to the Secretary at any time previous to April 25th.

---

POWDER FOR USE IN NASAL CATARRH—Beverly Robinson:

| | |
|---|---|
| Iodoform, porphyrized, | 4 |
| Camphor, powdered, | 4 |
| Gum arabic, powdered, | 8 |

Mix. For use several times in the day.

